# Perceived stress and coping strategies among Tunisian health sciences students

**DOI:** 10.1192/j.eurpsy.2026.11812

**Published:** 2026-07-31

**Authors:** H. Daoud, I. Sellami, A. Feki, M. Hajjaji, M. L. Masmoudi, K. Jmal Hammami

**Affiliations:** 1 Department of occupational medicine, Hedi Chaker Hospital; 2 LR/18/ES-28, Sfax university; 3 Department of rheumatology, Hedi Chaker Hospital, Sfax, Tunisia

## Abstract

**Introduction:**

Health sciences students are frequently exposed to academic pressure, clinical responsibilities, and emotional challenges, which can contribute to elevated stress levels. Coping strategies play a crucial role in how individuals manage stress and maintain well-being.

**Objectives:**

This study aimed to assess the relationship between stress levels and coping strategies among health sciences students.

**Methods:**

We conducted a cross-sectional study among health sciences students. Data were collected anonymously through a Google Forms survey from October 2023 to January 2024. Participants completed a questionnaire collecting sociodemographic and academic characteristics, followed by standardized assessments of stress and coping. Stress was measured using the 4-item Perceived Stress Scale (PSS-4). Coping strategies were evaluated with the Brief COPE Inventory, which distinguishes between problem-focused coping (PFC), emotion-focused coping (EFC), and avoidance coping (AC).

**Results:**

Our study included 472 health sciences students, with a sex ratio of 0.44. Nursing science students represented 71.8% of the sample. The mean of participants perceived stress was 7.19 ± 2.45. The mean total coping score among students was 63.97 ± 10.96, with subscale means of 19.54 ± 3.74 for problem-focused coping, 25.56 ± 4.50 for emotion-focused coping, and 17.83 ± 3.47 for avoidance coping. Bivariate analysis showed that higher stress level was associated with lower scores on emotion-focused coping (EFC) (r=-0.093, p=0.044) and avoidance coping (AC) (r =-0.132, p =0.004).

**Conclusions:**

Higher levels of perceived stress were inversely associated with the use of emotion-focused and avoidance coping strategies, underscoring the need to promote effective coping mechanisms to enhance students’ mental well-being.

**Disclosure of Interest:**

None Declared

